# Identification of Hub Genes Associated with Nonspecific Orbital Inflammation by Weighted Gene Coexpression Network Analysis

**DOI:** 10.1155/2022/7588084

**Published:** 2022-05-27

**Authors:** Hanhan Liu, Lu Chen, Xiang Lei, Hong Ren, Gaoyang Li, Zhihong Deng

**Affiliations:** ^1^Department of Ophthalmology, The Third Xiangya Hospital, Central South University, Changsha, Hunan, China; ^2^Department of Ophthalmology, Xiangya Hospital, Central South University, Changsha, Hunan, China

## Abstract

**Background:**

Nonspecific orbital inflammation is a common ophthalmopathy with a high prevalence among adult females. Yet, its molecular mechanisms behind are poorly understood. Regulation of gene expression probably plays an important role in this disease. Thus, we utilized gene coexpression networks to identify key modules and hub genes involved in nonspecific orbital inflammation.

**Methods:**

Data of gene expression in nonspecific orbital inflammation samples (*n* = 61) and healthy samples (*n* = 28) were obtained from the public Gene Expression Omnibus database. Afterward, differentially expressed genes were performed. Then, weighted correlation network analysis was done to define the key modules. Next, functional enrichment analysis was conducted by Gene Ontology and Kyoto Encyclopedia of Genes and Genomes pathway in key modules. Finally, a protein-protein interaction network and Cytohubba plugin were used to screen hub genes.

**Results:**

Differential expression of 716 genes was identified, among which 169 genes were upregulated and 547 genes were downregulated in the nonspecific orbital inflammation group. In weighted correlation network analysis, we clarified 2 key modules (MEturquoise and MEblue) that are likely to play key roles in nonspecific orbital inflammation. Functional enrichment analysis indicated that these genes are predominately involved in immune response and matrix homeostasis. In addition, among 2 key modules, there are 20 hub genes identified.

**Conclusion:**

With this new approach, we identified several genes that could be critical to pathologies of nonspecific orbital inflammation. These findings may contribute to further therapeutic target development.

## 1. Introduction

Nonspecific orbital inflammation (NSOI), an idiopathic chronic proliferative inflammatory disease, was first described in 1905 by Birch-Hirschfield [1]. It is also known as “idiopathic orbital inflammatory syndrome” or “orbital pseudotumor.” NSOI accounts for approximately 6%–16% of all orbital lesions and 11% of orbital tumors [2–4]. It is prevalent among middle-aged adults, especially females [5, 6]. The detailed pathophysiological cause for NSOI remains unknown. Some studies suggest it might be correlated with Streptococcal pharyngitis, viral upper respiratory infection, or other autoimmune disorders, such as rheumatologic disease, multifocal fibrosclerosis, and Crohn disease [7, 8]. The typical clinical feature of NSOI is an acute onset of orbital ache and headache, lid swelling, and proptosis with unilateral polymorphous lymphoid infiltration [5, 9]. For treatment, steroids are the standard therapeutics [10]. The recurrence rate is still higher than 50% even with proper corticosteroid treatment [11]. Thus, further understanding of the molecular mechanism of NSOI is essential for the development of novel therapeutic approaches to prevent recurrence as well as improve the outcomes of patients.

With the support of bioinformatic tools, high-throughput data analysis was widely used to systematically identify the functional networks of genes in different disease models, thus providing important clues for molecular mechanism studies [12, 13]. In NSOI-related studies, by using microarrays, Rosenbaum et al. have identified the gene expression profile NSOI [14]; moreover, they found there is no significant difference between granulomatosis with polyangiitis (GPA) and NSOI [15]. However, little is known about the hub genes which are closely related to the pathogenesis of NSOI. Herein, we used a novel tool, weighted gene coexpression network analysis (WGCNA), to identify the potential molecular interaction and correlation networks in this disease. WGCNA is an effective bioinformatic method to clarify synergetic expressed modules and to identify the relationship of gene networks at the transcriptome level [16]. It can provide high sensitivity to genes with low abundance or marginal fold changes [17]. In recent years, WGCNA has been successfully applied in different disease models to generate correlation networks, further identifying candidate biomarkers or therapeutic targets [18–21].

In our study, WGCNA was used to analyze the differentially expressed genes (DEGs) from 89 samples from a public dataset. Then, key gene modules related to DEGs were defined. We also examined the biological functions and pathways of genes in the key modules. These informative genes identified in our study may provide a novel insight into the understanding of the pathogenesis of NSOI. Moreover, the findings may be significant to new therapeutic target development for the treatment of NSOI patients.

## 2. Methods

### 2.1. Data Preparation and Preprocessing

The WGCNA dataset related to NSOI was downloaded from NCBI GEO (http://www.ncbi.nlm.nih.gov/geo) with accession number GSE58331, which consists of 89 samples (28 samples of normal control, 61 samples of NSOI). The (HG-U133_Plus_2) Affymetrix Human Genome U133 Plus 2.0 Array platform was used. Prior to WGCNA analysis, DEGs from the candidate genes were identified using the limma package in R from the Bioconductor website: (http://www.bioconductor.org/packages/release/bioc/html/limma.html). *P* < 0.05 and |log2 (fold change)| > 1 were considered the cut-off criteria.

### 2.2. Screening NSOI-Related Key Modules Based on WGCNA

WGCNA [16] is a typical biologic algorithm for constructing gene coexpression networks. We used the WGCNA to analyze the expression values of the DEGs obtained in the previous screening in each group and screened the modules and genes associated with NSOI. Firstly, to calculate the adjacency matrix, the trait-based node significance measure was calculated with the following formula:

(1) $$\begin{array}{c} S_{ij}=\left|{\text{cor}}_{\left(i,j\right)}\right|, \end{array}$$

where *i* and *j* stand for the expression gene *i* and *j*, respectively. The Pearson coefficient of these two vectors was defined as cor. To improve the robustness of the coexpression network, this transition was designed to give more weight to the strong connections. Meanwhile, we decreased the value of weak connections in the predicted coexpression network.

Subsequently, a *power function* was then applied to correlate the adjacency of genes:

(2) $$\begin{array}{c} a_{ij}={\text{power}}_{\left(S_{ij},\beta \right)}. \end{array}$$

Afterward, the adjacency matrix was converted to a topological matrix using the following formula:

(3) $$\begin{array}{c} w_{ij}=\frac{l_{ij}+a_{ij}}{\min\left\{k_{i},k_{j}\right\}+1-a_{ij}}. \end{array}$$

The topological properties were also confirmed. Then, the dynamic tree cut method was used to accomplish module identification. Highly similar modules were identified by cluster analysis and then merged with a height cut-off of 0.95. Furthermore, the *p* value of gene expression difference between the NSOI group and control was evaluated with a Student *t*-test. The significant gene was defined by the log *P* value. The mean value of gene significance (GS) derived from modules comprising gene was defined as module significance (MS).

### 2.3. Functional Enrichment Analysis in Key Modules

To understand the functional significance of DEGs in NSOI-related key modules, the Gene Ontology (GO) enrichment analysis and Kyoto Encyclopedia of Genes and Genomes (KEGG) pathway enrichment analysis were performed by using the R package. We used DAVID 6.7 (https://david-d.ncifcrf.gov/) online tools to conduct the GO analysis. GO enrichment analysis consists of cellular component (CC), molecular function (MF), and biological process (BP). The cut-off of *P* value < 0.05 was regarded as significant.

### 2.4. Protein-Protein Interaction (PPI) Network Analysis

The PPI network of DEGs was established using the Search Tool called TRING [22] (Version: 10.0, http://www.string-db.org/) to predict and analyze the interactions between proteins encoded by DEGs. In the network, nodes represent genes and edges represent the interactions between the nodes. Then, the software Cytoscape (Cytohubba plugin) [23] (Version: 3.2.0, http://www.cytoscape.org/) was used to perform the network analysis.

### 2.5. Identification of Hub Genes

Based on the PPI network, the software Cytoscape (Cytohubba plugin) [23] (Version: 3.2.0, http://www.cytoscape.org/) was used to perform the network analysis. The molecular Complex Detection algorithm was used within Cytoscape to detect crucial gene clusters based on the DEG coexpression network. The top 10 of the high degree genes in each module were identified based on the MCC method.

## 3. Results

### 3.1. Identification of DEGs between NSOI and Healthy Controls

**Figure 1:**
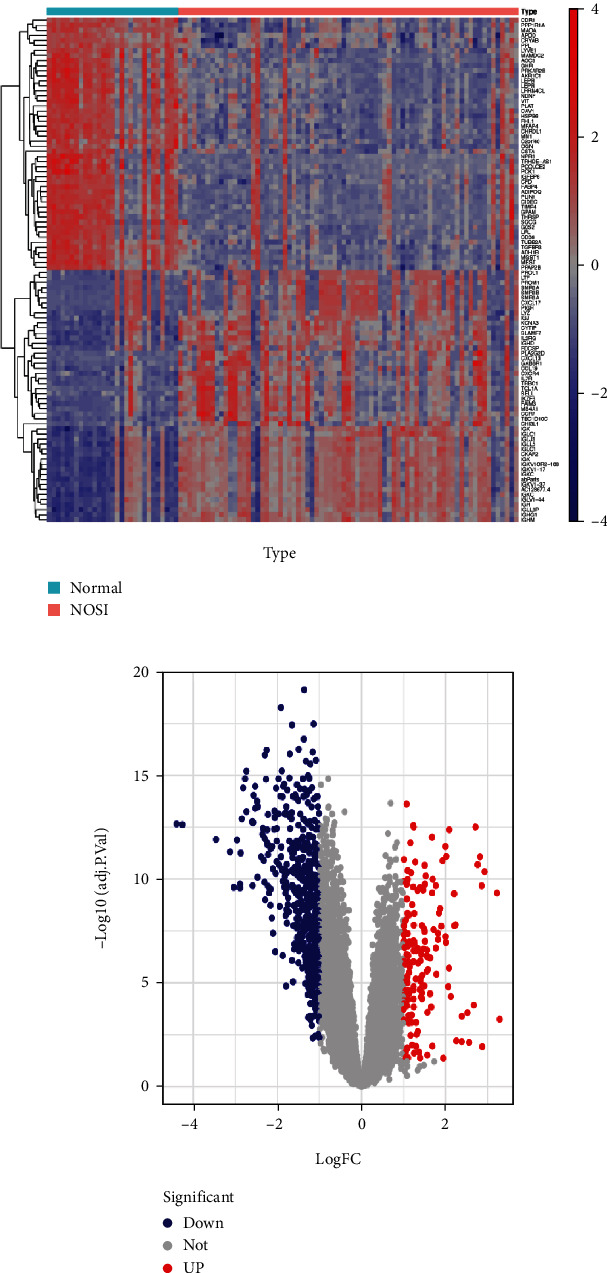
The heatmap (a) and volcano plot (b) of DEGs between NSOI and healthy controls.

To identify the DEGs, the gene microarray data of 89 samples (28 samples of normal control and 61 samples of NSOI) were downloaded from the GEO database. After normalization, batch correction, and gene annotation, gene expression distribution of each sample was depicted. Subsequently, a total number of 716 DEGs were identified, of which 169 genes were upregulated and 547 genes were downregulated in the NSOI group. The gene expression heatmap and volcano plot are shown in Figures 1(a) and 1(b), respectively.

For bars on the top, the light blue bar indicates a normal sample, while the light red bar indicates the NSOI sample.

### 3.2. Identification of Key Modules Based on WGCNA

**Figure 2:**
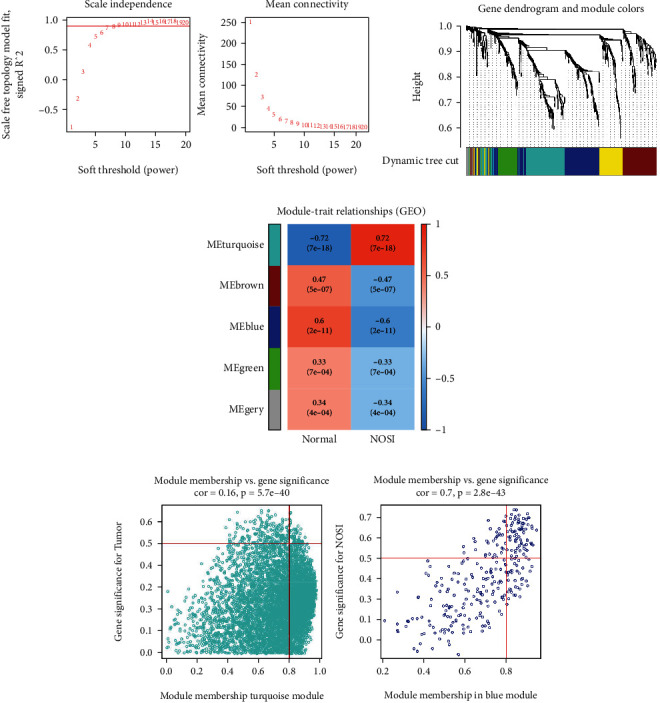
Identification of key modules based on WGCNA: (a) analysis of the scale-free topology model fit index for soft threshold powers (*β*) and the mean connectivity for soft threshold powers. (A) Displays the influence of soft-thresholding power (*x*-axis) on a scale-free fit index (*y*-axis). (B) Shows the influence of soft-thresholding power (*x*-axis) on mean connectivity (degree, *y*-axis). The approximate scale-free topology can be attained at the soft-thresholding power of 8. (b) A cluster dendrogram was built based on the dissimilarity of the topological overlap, together with assigned module colors. (c) Heatmaps of the plot of the adjacencies in the hub gene network include the trait weight. (d) The scatter plots of gene significance (GS) versus Module Membership (MM) of MEturquoise and MEblue.

To better understand the gene expression network of NSOI, WGCNA was performed on the obtained 716 DEGs. Firstly, network topology analysis was performed to obtain relatively balanced scale independence and mean connectivity of the WGCNA. As shown in Figure 2(a), the *X*-axis shows matrix weighting power while the *Y*-axis shows a quadratic correlation index derived from log (*k*) and log (*P*(*k*)) of the corresponding network. In this figure, when the correlation index reached 0.90, we took power as 8. Subsequently, a hierarchical clustering tree (dendrogram) of the 716 genes was analyzed (Figure 2(b)). Then, five modules (MEturquoise, MEbrown, MEblue, MEgreen, and MEgrey) were generated with the setting of MEDissThres as 0.25 (Figure 2(c)), of which MEturquoise was identified as the key module which has the strongest positive correlation with trait weight of NSOI, while MEblue has the strongest negatively correlation with trait weight of NSOI. The scatter plots of gene significance (GS) versus Module Membership (MM) of MEturquoise and MEblue are shown in Figure 2(d). Taken together, MEturquoise and MEblue were considered to be the key modules in the NSOI. Both of them were taken into further study.

### 3.3. Functional Enrichment Analysis of Genes in MEturquoise and MEblue

**Figure 3:**
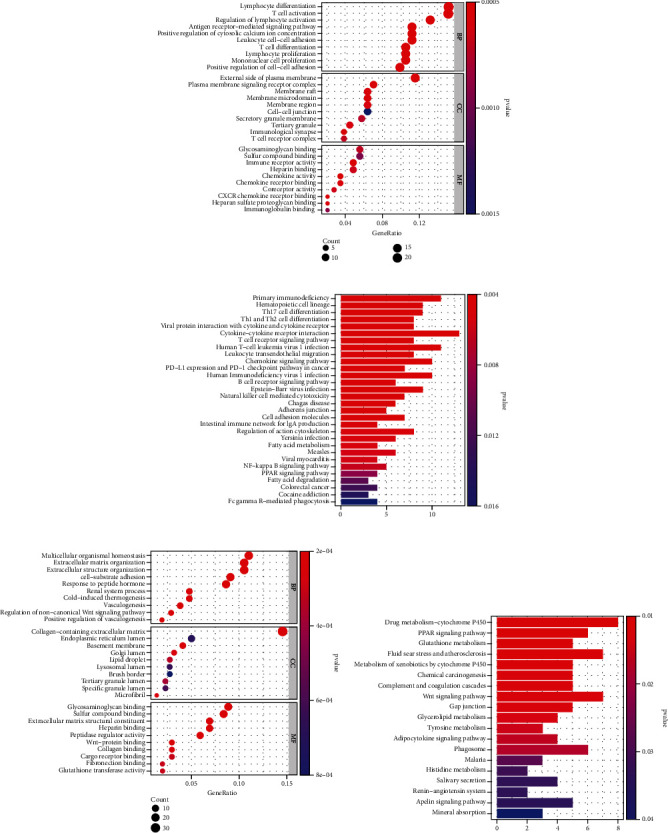
Functional enrichment analysis of genes in MEturquoise and MEblue by GO and KEGG analyses. (a) Bubble plot showed results of GO analysis (BP, CC, and MF) in MEturquoise module. (b) Barplot showed KEGG analysis of genes in MEturquoise module. (c) GO analysis (BP, CC, and MF) of genes in MEblue. (d) KEGG analysis of genes in MEblue.

To clarify the biological functions associated with NSOI in the MEturquoise, functional enrichment analyses including GO and KEGG pathway enrichment analyses were performed. For GO biological processes, genes in MEturquoise were significantly enriched in “lymphocyte differentiation,” “T cell activation,” and “lymphocyte activation” (Figure 3(a)). For KEGG pathway analysis, the genes were mainly enriched in “cytokine−cytokine receptor interaction” and “primary immunodeficiency” (Figure 3(b)). In genes of MEblue, the GO analysis revealed that the most significant GO terms were “organismal homeostasis,” “extracellular matrix,” and “cell−substrate adhesion” (Figure 3(c)). The KEGG pathway analysis showed that the most significantly enriched pathways were “drug metabolism−cytochrome P450,” “fluid shear stress and atherosclerosis,” and “Wnt signaling pathway.” (Figure 3(d)) Pathway analysis suggested that local inflammation was involved in the development of NSOI.

### 3.4. PPI and Coexpression Networks to Identify Hub Genes Associated with NSOI

**Figure 4:**
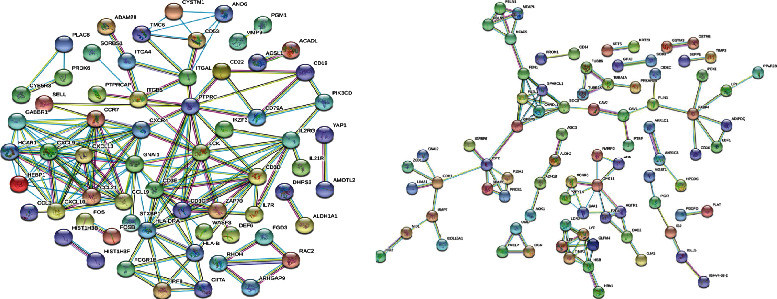
PPI network analysis in MEbrown (a) and MEblue (b).

**Figure 5:**
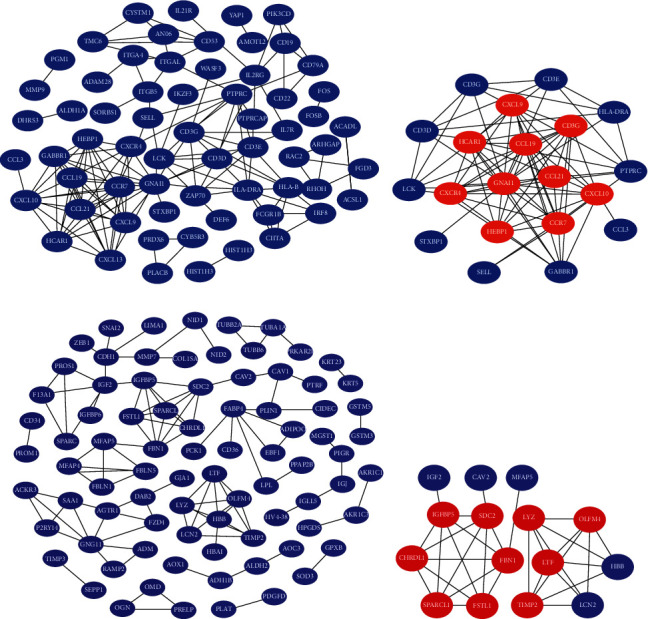
Hub gene identification in MEturquoise and MEblue. Genes labeled with red were considered hub genes.

Subsequently, PPI network analysis was used to predict and analyze the interactions between proteins encoded by DEGs in MEturquoise and MEblue. As shown in Figures 4(a) and 4(b), for the whole network, there were 152 nodes in the MEturquoise and 105 nodes in the MEblue. As highly connected genes in the key modules (MEturquoise and MEblue), they play significant parts in the biological processes of NSOI. We chose the top 10 genes ranked by degree of the protein-protein interaction nodes as candidate hub genes (Table 1). In MEturquoise, identified hub genes were GNAI1, CXCR4, CCR7, CXCL10, CCL21, CCL19, CXCL13, CXCL9, HEBP1, and HCAR1 (Figures 5(a) and 5(b)), and in MEblue, hub genes include SDC2, IGFBP5, FBN1, FSTL1, CHRDL1, SPARCL1, LYZ, LTF, OLFM4, and TIMP2 (Figures 5(c) and 5(d)).

The node size is based on the PPI degree value.

## 4. Discussion

In recent years, the high-throughput sequencing technique generates huge data for biological studies. Until now, the majority of the existing bioinformatic tools concentrate only on unweighted networks [14]. However, WGCNA is a comprehensive novel statistical approach that can be used for both weighted and unweighted correlation networks and to further explore the module (cluster) structure in a network. Moreover, it also can be used to rank genes or modules in independent datasets to identify the hub genes [16]. In our study, 2 modules including the turquoise module (MEturquoise) and the blue module (MEblue) were considered to be the key modules in NSOI by using WGCNA. GO and KEGG pathway enrichment analyses were performed on genes in these two modules and identify that inflammation and immune-related pathways are broadly involved in NSOI.

The development of NSOI is a complex, heterogeneous, and multifactorial process [24]. In the present study, the results from GO and KEGG pathway analyses suggested that immune cells, including lymphocyte and T cells, were closely associated with the pathogenesis of NSOI. Moreover, the imbalance of cellular homeostasis and dysfunction of the extracellular matrix could be the other molecular mechanism of NSOI development. Previous studies suggested that aberrant immune-mediated production of fibrogenic cytokines leads to the progression of NSOI [25, 26]. Followed studies have focused on NSOI-related immunophenotypic features. Lowen et al's [27] study enrolled 55 cases for exploring the histopathologic and immunohistochemical pattern of NSOI; they found that NSOI displayed a predominance of T cells, which coordinates with our results. Despite the immune alteration, variable degrees of collagen deposition also are considered an essential pathologic change of NSOI as previously reported [28, 29]. However, the cellular mechanism remains not fully understood.

PPI networks are useful in cell functions and disease mechanism prediction, especially for relationships between the species through conserved pathways and protein complexes, and discover new therapeutic targets [30, 31]. In this study, based on the DEGs in MEturquoise and MEblue, we identified 152 nodes in the MEturquoise and 105 nodes in the MEblue in the whole PPI network. Then, hub genes in each module were identified based on the Cytohubba degree. The hub genes include GNAI1, CXCR4, CCR7, CXCL10, CCL21, CCL19, CXCL13, CXCL9, HEBP1, HCAR1, SDC2, IGFBP5, FBN1, FSTL1, CHRDL1, SPARCL1, LYZ, LTF, OLFM4, and TIMP2. Among them, GNAI1, which is known as G protein subunit alpha i1, has the highest connection degree. GNAI1 is a member of the GNA family which is abundantly expressed in immune cells; it mainly participates in G protein-coupled receptor (GPCR) and non-GPCR signaling pathways [32, 33]. Studies reported that GNAI1 may have effects on angiogenesis by regulating VEGF-induced Akt-mTOR and Erk-MAPK activation [34]. Moreover, GNAI1 could act as a tumor suppressor in colon cancer by regulating the IL6 signaling pathway [35]. The genes of chemokines and chemokine receptors, such as CXCR4, CCR7, CXCL10, CCL21, CCL19, CXCL13, and CXCL9, were enriched in NSOI tissue, suggesting that chemokine-related factors may play important roles in the pathologic process of NSOI. Recently, studies demonstrated that chemokine-related pathways, e.g., IGF-1R and PPAR*γ* signaling pathways, were involved in NSOI [36, 37]. However, the mechanism of GNAI1 and chemokines in NSOI is insufficient and needs further investigation.

In summary, with WGCNA network analysis, our study identifies a coexpression module in genes of patients with NSOI. Two modules (MEturquoise and MEblue) were found highly enriched in multiple pathways. Furthermore, the identified 20 hub genes have the potential to be biomarkers for the diagnosis and treatment of NSOI.

## Figures and Tables

**Table 1:** Top 10 in network string ranked by MCC method in MEturquoise and MEblue.

Ranked by MCC	MEturquoise		MEblue	
	Gene name	Score	Gene name	Score
1	GNAI1	3628923.0	SDC2	121.0
2	CXCR4	3628804.0	IGFBP5	121.0
3	CCR7	3628801.0	FBN1	121.0
4	CXCL10	3628801.0	FSTL1	120.0
5	CCL21	3628800.0	CHRDL1	120.0
6	CCL19	3628800.0	SPARCL1	120.0
7	CXCL13	3628800.0	LYZ	48.0
8	CXCL9	3628800.0	LTF	48.0
9	HEBP1	3628800.0	OLFM4	48.0
10	HCAR1	3628800.0	TIMP2	48.0

## Data Availability

The data that support the findings of this study are openly available in GEO (http://www.ncbi.nlm.nih.gov/geo), GSE58331.
